# Machine learning of native T1 mapping radiomics for classification of hypertrophic cardiomyopathy phenotypes

**DOI:** 10.1038/s41598-021-02971-z

**Published:** 2021-12-08

**Authors:** Alexios S. Antonopoulos, Maria Boutsikou, Spyridon Simantiris, Andreas Angelopoulos, George Lazaros, Ioannis Panagiotopoulos, Evangelos Oikonomou, Mikela Kanoupaki, Dimitris Tousoulis, Raad H. Mohiaddin, Konstantinos Tsioufis, Charalambos Vlachopoulos

**Affiliations:** 1 Unit of Inherited Cardiac Conditions and Sports Cardiology, 1st Department of Cardiology, National and Kapodistrian University of Athens, Athens, Greece; 2 CMR Unit, Mediterraneo Hospital, Attiki, Greece; 3 CMR Unit, Faculty of Medicine, National Heart and Lung Institute, Imperial College London, London, UK

**Keywords:** Diagnostic markers, Cardiology

## Abstract

We explored whether radiomic features from T1 maps by cardiac magnetic resonance (CMR) could enhance the diagnostic value of T1 mapping in distinguishing health from disease and classifying cardiac disease phenotypes. A total of 149 patients (n = 30 with no heart disease, n = 30 with LVH, n = 61 with hypertrophic cardiomyopathy (HCM) and n = 28 with cardiac amyloidosis) undergoing a CMR scan were included in this study. We extracted a total of 850 radiomic features and explored their value in disease classification. We applied principal component analysis and unsupervised clustering in exploratory analysis, and then machine learning for feature selection of the best radiomic features that maximized the diagnostic value for cardiac disease classification. The first three principal components of the T1 radiomics were distinctively correlated with cardiac disease type. Unsupervised hierarchical clustering of the population by myocardial T1 radiomics was significantly associated with myocardial disease type (chi^2^ = 55.98, p < 0.0001). After feature selection, internal validation and external testing, a model of T1 radiomics had good diagnostic performance (AUC 0.753) for multinomial classification of disease phenotype (normal vs. LVH vs. HCM vs. cardiac amyloid). A subset of six radiomic features outperformed mean native T1 values for classification between myocardial health vs. disease and HCM phenocopies (AUC of T1 vs. radiomics model, for normal: 0.549 vs. 0.888; for LVH: 0.645 vs. 0.790; for HCM 0.541 vs. 0.638; and for cardiac amyloid 0.769 vs. 0.840). We show that myocardial texture assessed by native T1 maps is linked to features of cardiac disease. Myocardial radiomic phenotyping could enhance the diagnostic yield of T1 mapping for myocardial disease detection and classification.

## Introduction

Cardiac magnetic resonance (CMR) is considered the state-of-the-art imaging approach for assessing myocardial disease^1^, allowing tissue characterization and fibrosis detection by late gadolinium enhancement (LGE)^2^. Myocardial T1 mapping is also helpful across a spectrum of disease conditions, including diffuse interstitial or replacement fibrosis, water, or infiltrative disorders^2,3^. However, a major problem with T1 mapping is that there is a significant overlap in myocardial native T1 values between health and disease; thus, while in-patient regional or even temporal variations in native T1 may be informative of disease development or progression, between-patient comparisons are less useful. A method that could harness the richness of information contained in myocardial T1 maps, would enhance the diagnostic value of T1 mapping for rapid detection of myocardial disease without the need of contrast agents.

Radiomics is a rapidly evolving field, which uses data-characterization algorithms to extract data from medical images^4^. The segmented anatomical volumes are rendered into quantitative radiomic features that provide information on tissue volume, shape, and texture by analyzing the spatial relationship of (dis)similar voxels (an analogous to terrain mapping)^5–8^. In other medical fields, such as in clinical oncology, radiomic phenotyping has been successfully used to characterize the distinct biological phenotypes of tumors and provide relevant prognostic information^7,8^. More recently, we have also shown how pericoronary tissue CT radiomics can enhance cardiovascular risk stratification^4^. Since T1 mapping is a standardized CMR acquisition, extraction of radiomic features by myocardial T1 maps is feasible^9^ and recent evidence supports their value for classification between hypertensive heart disease and hypertrophic cardiomyopathy (HCM)^10^. However more evidence is needed to fully establish the clinical value of T1 radiomics in reliably discriminating between various cardiac phenotypes.

The aim of this proof-of-concept study was to extract and validate radiomic features from T1 maps using machine learning in a wide spectrum of conditions ranging from normal to various myocardial diseases with particular emphasis on distinguishing healthy from diseased myocardium and classifying LVH phenotypes.

## Material and methods

### Study population

*Study Arm 1* comprised of 20 CMR scans with available native T1 mapping randomly selected by our archive (CMR scan performed for various clinical indications). These imaging datasets served for the purpose of stability assessment (i.e*.*, inter-observer variability) of extracted radiomic features from T1 maps, which were included in further analysis in Study Arm 2.

*Study Arm 2* included a total of 152 consecutive patients undergoing a CMR scan (period 2019–2020) and native T1 mapping as follows: individuals without evidence of structural heart disease on CMR (n = 30), patients with left ventricular hypertrophy (LVH, i.e. increased wall thickness ≥ 12 mm or increased LV mass index) of various causes (athletes, valvular heart disease, hypertension etc., n = 30), patients with known HCM (n = 61) and patients with known cardiac amyloidosis (n = 28). Three patients (n = 3) with suboptimal image quality of T1 maps (i.e., breathing artefacts) were excluded from analysis. The study was approved by the Institutional Research Ethics Committee (Hippokration General Hospital of Athens) with waiver of consent since the study involved only the use of anonymized imaging datasets; no individual patient data or human tissue samples were collected. All research was performed in accordance with relevant guidelines/regulations and in accordance with the Declaration of Helsinki.

### Study design

**Figure 1:**
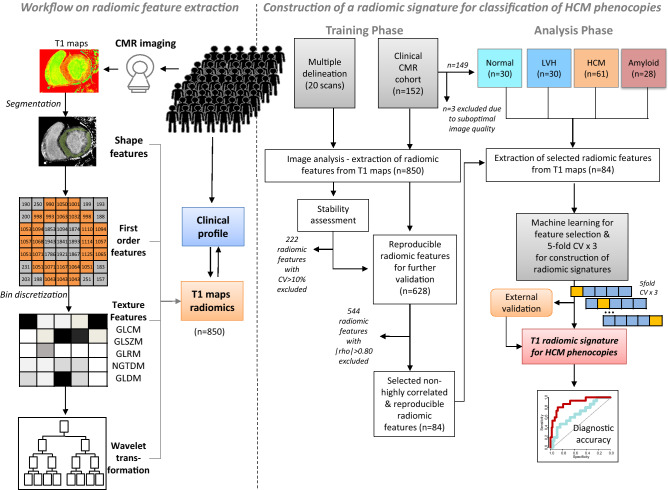
Study flow-chart. *CMR* cardiac magnetic resonance, *CV* coefficient of variation, *GLCM* gray level co-occurrence matrix, *GLDM* Gray Level Dependence Matrix, *GLRM* Gray Level Run-Length Matrix; *GLSZM* Gray Level Size Zone Matrix, *HCM* hypertrophic cardiomyopathy; *LVH* left ventricular hypertrophy; *NGTDM* Neighboring Gray Tone Difference Matrix.

**Figure 2:**
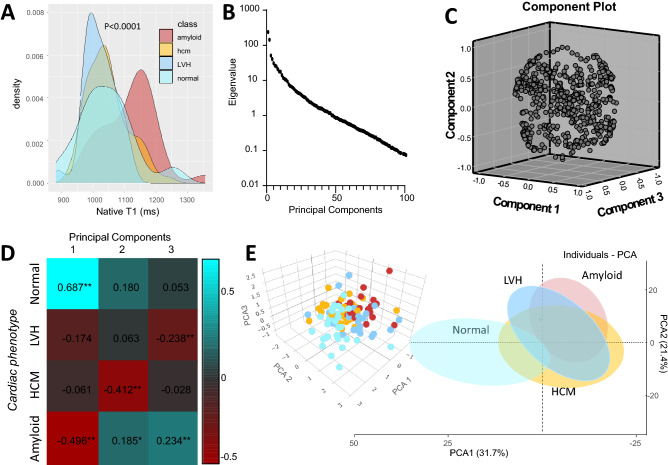
(**A**) Density plots for myocardial native T1 values for the population subgroups. (**B**) Dotplot of the eigenvalue vs principal components of T1 radiomics. (**C**) Cloud plot of the first three principal components of T1 radiomics for the observation of the whole study population (Arm 2) and (**D**) correlations with cardiac phenotypes. (**E**) Cloud plot of the first three principal components (PCAs) for the observations of Arm 2 colored by disease background (right) and two-dimensional scatterplot of the first two PCAs with the ellipsoid shaded areas denoting the 95% confidence intervals for the observations included in each subgroup. *HCM* hypertrophic cardiomyopathy, *LVH* left ventricular hypertrophy.

A schematic of the study flowchart is provided in Fig. 1. The objective of the study was to perform feature selection of T1 radiomics by machine learning to identify biomarkers of disease that could be used for classification of cardiac phenotypes and demonstrate their incremental diagnostic value compared to the native T1 values (Fig. 1 left). To achieve this, we followed a stepwise approach. First, in a randomly selected population from our archive (n = 20), we assessed the stability of extracted radiomic features from T1 maps (Study Arm 1, see “Study population”, Fig. 1 right). Subsequently, in a proof-of-concept analysis, we demonstrated the association of cardiac health/disease phenotypes with the 3 main principal components of radiomic features in our main study population (n = 149, Study Arm 2 (Fig. 2). Then, we explored the diagnostic information of the whole available radiomic dataset by performing unsupervised clustering of the population and by showing how formed patient clusters based on population radiomic features differ in the prevalence of cardiac disease phenotypes (Figs. 1 right, 3). Next, we applied a machine learning algorithm to eliminate highly correlated features and kept only those radiomic features that were stable and non-highly correlated as potential biomarkers of interest. Their value in multinomial classification was internally (supervised classification) and externally tested and the most important predictors were identified by a random forest Machine Learning algorithm (Fig. 4). Their diagnostic performance over and above native T1 for cardiac phenotype classification was demonstrated in relevant ROC curves (Figs. 1 right, 5).

**Figure 3:**
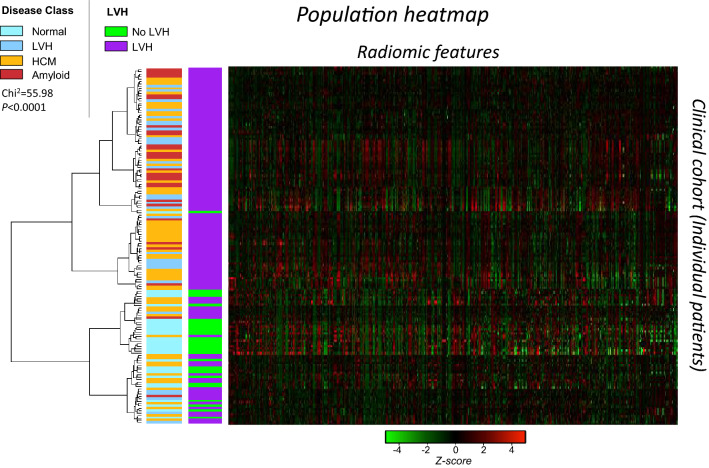
Unsupervised hierarchical clustering of patients of Arm 2 by T1 radiomics (n = 628) and resulting heat map with a row dendrogram indicating the patient clustering. Vertical colored legends on the left of the heat map indicate the cardiac phenotype for each observation (patient). *HCM* hypertrophic cardiomyopathy, *LVH* left ventricular hypertrophy. Chi^2^ p value is reported for the difference in disease class between the two parent clusters.

**Figure 4:**
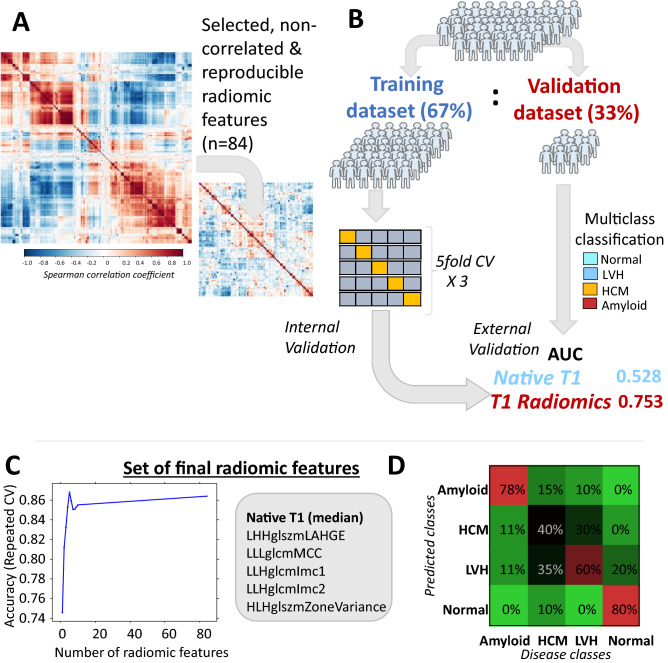
(**A**) Correlation plot of the stable T1 radiomic features (n = 628) and resulting clustering in groups of highly correlated features based on spearman’s rho coefficient and resulting correlation plot after stepwise exclusion of highly correlated radiomic features (n = 84) which were subsequently tested for classification of cardiac phenotype. (**B**) The population of Arm 2 was split in a training (67%) and validation (33%) dataset for model training and validation, respectively. The 84 stable and non-highly correlated T1 radiomic features were entered in multinomial models trained with fivefold cross-validation repeated 3 times, which were then tested in validation dataset. (**C**) A set of the most important radiomic features that maximized model’s accuracy were finally selected. (**D**) Confusion matrix for predicted vs observed classes with the use of the final radiomic model in the testing dataset. *AUC* area under curve, *CV* coefficient of variation, *HCM* hypertrophic cardiomyopathy, *LVH* left ventricular hypertrophy.

**Figure 5:**
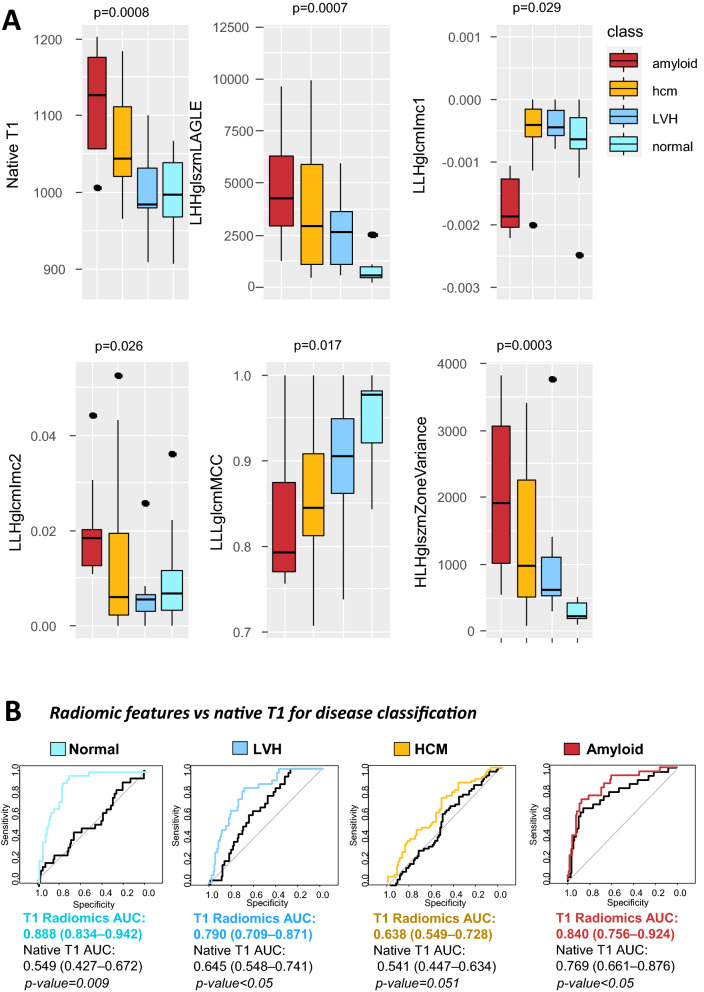
(**A**) Boxplots showing the distribution of the finally selected radiomic features in the testing cohort between disease classes. p values are derived from Kruskal–Wallis. (**B**) The incremental value of the final radiomic features for disease classification vs. native T1 (mean) values was demonstrated in relevant receiver operating characteristic curves.

### Cardiac magnetic resonance scans

All CMR scans were performed on an Ingenia 1.5 T MR system (Philips Healthcare). Optimized single breath hold T1 Modified Look -Locker Inversion Recovery (MOLLI) sequence using the native 5 s(3 s)3 s scheme was used for T1 mapping with the following acquisition settings: preset FOV 320 × 320 mm, slice thickness 10 mm, Relative SNR 1.0, TR/TE 2.3/1.08, TFE factor 80 ACQ Matrix 160 × 160. As per our local clinical practice T1 mapping was routinely used in all patients with well controlled resting heart rate i.e., < 80–85 bpm. Also, patients with T1 mapping of suboptimal quality (i.e., breath-hold artefacts) were not included in the final study population (n = 3).

### Extraction of myocardial radiomic features from native T1 maps

Extraction of radiomic features from left ventricular (LV) myocardium of native T1 maps was done using the 3D Slicer software (v.4.9.0-2017-12-18 r26813, available at http://www.slicer.org)^11^. Operators were blinded to the disease status of patients and were trained and supervised in T1 mapping analysis by an experienced CMR Level 3 accredited individual. Segmentation of LV myocardium was performed on basal LV short axis slice. LV myocardium was manually segmented with manual offset of epicardial and endocardial layers to avoid contamination by epicardial fat or blood pool, respectively. The segmented volume of interest was subsequently used to calculate and extract a series of radiomic features, using the SlicerRadiomics extension of 3D Slicer, which incorporates the Pyradiomics library into 3D Slicer^12^. Shape-related and first-order radiomic features were calculated using the native T1 values of the segmented myocardium. For calculation of texture features i.e., Gray Level Co-occurrence Matrix (GLCM), Gray Level Dependence Matrix (GLDM), Gray Level Run-Length Matrix (GLRLM), Gray Level Size Zone Matrix (GLSZM), and Neighboring Gray Tone Difference Matrix (NGTDM), voxels of LV myocardium were discretized into 16 bins of equal width, to reduce noise while allowing a sufficient resolution to detect biologically significant spatial changes in native T1 myocardial values^6,13^. To enforce symmetrical, rotationally-invariant results, texture statistics (GLCM etc.) were calculated in all four directions and then averaged, as previously described^6^.

#### Wavelet transformation

First-order and texture-based statistics were also calculated for wavelet transformations of the original image resulting in eight additional sets of radiomic features^14^. In particular, wavelet transformation decomposes the data into high and low-frequency components. At high frequency (shorter time intervals), the wavelets can capture discontinuities, ruptures and singularities in the original data. At low frequency (longer time intervals), the wavelet characterizes the coarse structure of the data to identify the long-term trends. Thus, the wavelet analysis allows extraction of hidden and significant temporal features of the original data, while improving the signal-to-noise ratio of imaging studies^7^.

### Machine learning and statistical analysis

#### Feature selection and stability assessment

In order to limit our analysis to radiomic features that could be of value as imaging biomarkers, we performed a stability assessment of all extracted radiomic features. For this purpose, we used 20 scans from Study Arm 1 to assess the coefficient of variation (CV) of each radiomic feature in multiple delineations by two independent operators. Only those radiomic features with multiple delineation CV < 10% were included in further analysis (n = 628, Fig. 1 right).

#### Principal components analysis

Calculated LV myocardium radiomic features were included in principal component analysis to identify principal components (PC) that describe most of the phenotypic variation in the study population. The first two components (PC1, PC2) were used to explore associations with disease background in relevant cluster plots (Fig. 2).

#### Unsupervised clustering of the study population by T1 radiomic features

The 628 selected radiomic features of LV of the patients of Study Arm 2 were transformed to Z-scores for further analysis. Then all 628 stable radiomic features were used to perform hierarchical clustering of the observations (i.e. patients) using the Ward method and the squared Euclidean distance (*hclust* R package). The variation of each of the 628 different radiomic features across the n = 149 observations of the Study Arm 2 cohort was represented in a relevant heat map with a row dendrogram indicating the clustering of patients (Fig. 3). The relationships between the 628 stable radiomic features were inspected in a corplot. Next a stepwise approach was applied and highly intercorrelated radiomic features (|rho|> 0.80) were removed from further analysis by application of a widely used automated algorithm^15^. This function (function *caret::findCorrelation*, R package) searches through a correlation matrix and returns a vector of integers corresponding to columns to remove and reduce pair-wise correlations. The absolute values of pair-wise correlations are considered. If two variables have a high correlation, the function looks at the mean absolute correlation of each variable and removes the variable with the largest mean absolute correlation (Fig. 4).

#### Machine-learning for classification of cardiac phenotype

We then attempted to select certain radiomic features as imaging biomarkers to describe heart disease phenotype and the presence of cardiac amyloidosis. The original population of Study Arm 2 was split into a training (67%) and a validation (n = 33%) dataset. Then the final 84 T1 radiomic features were included in multinomial logistic regression models (nnet::multinom) to seek independent associations with the cardiac disease phenotype (normal, LVH, HCM or cardiac amyloid). Machine learning was used for the internal cross-validation of the filtered radiomic features and final feature selection. A random forest algorithm (*caret* package R) with fivefold cross-validation repeated 3 times was used to select the top features able to classify cardiac disease phenotype. Finally, the diagnostic performance of the top radiomic features identified from this validation process for classification of each cardiac disease phenotype was assessed by the c-index and compared to that of the native T1 values. R statistical package version 3.6.0 (https://www.R-project.org/)^16^ was used for all statistical analysis.

## Results

### Radiomic feature extraction

The native T1 values (mean) between patient subgroups are shown in Fig. 2A and Table 1. There was a significant overlap between healthy individuals, patients with LVH or HCM; only patients with cardiac amyloid had significantly higher native T1 values than the rest patient subgroups, with a median value of 1117 ms; however, this meant that even in cardiac amyloid, the T1 values of approximately a quarter of cardiac amyloid patients were overlapping with other cardiac conditions. We calculated a total of 850 radiomic features by segmentation of LV myocardium, which included 15 shape-related features, 18 first order statistics, 15 GLCM, 18 GLDM, 16 GLRLM, 16 GLSZM, and 5 NGTDM features as well as eight wavelet transformations for each one of them.

**Table 1:** xxx.

	Overall	Normal	LVH	HCM	Amyloid	P value
	n = 149	n = 30	n = 30	n = 61	n = 28	
Age, years	54.3 (17.9)	37.57 (16.8)	54.5 (16.18)	55.5 (14.5)	69.9 (12.0)	< 0.001
Male sex, n (%)	90 (60.4)	10 (33.3)	21 (70.0)	41 (67.2)	18 (64.2)	0.007
Height, cm	170.9 (9.6)	169.6 (9.2)	170.5 (11.2)	172.6 (9.9)	169.4 (6.8)	0.371
Weight, kg	79.7 (16.3)	68.5 (12.0)	88.6 (19.6)	81.8 (15.1)	77.3 (11.4)	< 0.001
Body mass index, kg/m^2^	27.1 (4.69)	23.75 (3.74)	30.62 (5.5)	27.20 (3.89)	26.83 (3.56)	< 0.001
Body surface area, m^2^	1.93 (0.23)	1.79 (0.19)	2.03 (0.28)	1.97 (0.22)	1.90 (0.16)	< 0.001
MWT, mm	14.0 (4.5)	7.7 (1.1)	12.7 (1.8)	17.2 (3.8)	15.1 (2.2)	< 0.001
LVEF, %	64.3 (11.9)	64.7 (4.6)	61.5 (13.8)	69.1 (9.5)	55.9 (15.1)	< 0.001
LVEDV, ml	150.8 (55.0)	137.8 (27.0)	183.5 (82.8)	141.5 (40.7)	149.9 (56.4)	0.002
LVEDVi, ml/m^2^	78.0 (26.7)	76.7 (11.4)	91.0 (42.1)	72.1 (18.5)	78.5 (29.0)	0.016
LVESV, ml	57.0 (38.9)	48.7 (14.0)	75.1 (53.6)	45.8 (27.5)	71.4 (49.4)	0.001
LVESVi, ml/m^2^	29.6 (20.1)	27.3 (6.2)	37.6 (29.3)	23.2 (13.5)	37.5 (25.0)	0.001
LV mass, g	135.7 (65.4)	76.1 (22.4)	142.3 (59.9)	154.7 (69.4)	151.6 (56.9)	< 0.001
LVMI, g/m^2^	73.3 (51.5)	59.4 (97.8)	72.5 (26.2)	77.9 (32.0)	79.5 (30.2)	0.386
LGE, n (%)	93 (62.4)	0 ( 0.0)	12 ( 40.0)	56 (91.8)	28 ( 100)	< 0.001
LGE (+) AHA segments	4.7 (5.6)	0.0 (0.0)	1.9 (3.6)	5.5 (4.0)	12.2 (6.1)	< 0.001
**HCM phenotype**						
Apical	–	–	–	9 (14.7)	–	
Concentric	–	–	–	6 ( 9.8)	–	
Localised basal septum	–	–	–	2 ( 5.4)	–	
Reverse curvature septal	–	–	–	44 (72.1)	–	
Native T1, ms	1041 [992–1099]	1021 [986–1048 ms]	1019 [984–1051]	1036 [993–1078]	1117 [1055–1170]	< 0.001

*AHA* American Heart Association, *HCM* hypertrophic cardiomyopathy, *LGE* late gadolinium enhancement, *LV* left ventricle, *LVEDVi* left ventricular end diastolic volume index, *LVEF* left ventricular ejection fraction, *LVESVi* left ventricular end systolic volume index, *LVH* left ventricular hypertrophy, *LVMI* left ventricular mass index, *LVSVi* left ventricular stroke volume index, *MWT* maximal wall thickness;

### Principal component analysis

Initially, before looking into associations of specific radiomic features with cardiac disease, we performed exploratory data analysis by reducing the original radiomic dataset of possibly correlated features to its principal components. A total of 32 components accounted for the 99.5% of variation in the study population (scree plot, Fig. 2B), while the first three components explained 55% of the observed variation (Fig. 2C). The first three components were variably associated with the underlying cardiac phenotype (Fig. 2D), suggesting that native T1 maps of LV myocardium contain rich extractable information associated with distinct phenotypes of human heart, which could be used to distinguish healthy myocardium from myocardial disease (Fig. 2E).

### Unsupervised clustering of patients based on the radiomic phenotyping of myocardium

Since principal components are inherent to the sample population studied and not of transferrable value as quantifiable biomarkers, we focused on the analysis of the radiomic features per se. From the initial pool of 850 measured radiomic features, we performed a stability assessment and calculated the CV for multiple delineation (Arm 1, Fig. 1). Only those radiomic features with CV < 10% (n = 628) were included in further analyses (Figure S1; Table S1).

This set of 628 radiomic features of myocardial T1 maps was then used to perform unsupervised hierarchical clustering of the population in Arm 2. Hierarchical clustering identified distinct clusters of patients, which significantly differed in the prevalence and type of cardiac disease (p < 0.001, Fig. 3), suggesting that the individual radiomic features are also useful to classify cardiac phenotype.

### Machine learning for identification and validation of individual T1 radiomic features for cardiac disease classification

Having demonstrated the proof-of-concept that the radiomic features of myocardial T1 maps are linked with cardiac disease phenotype, we next attempted to identify specific radiomic features that could be used as biomarkers of cardiac disease. In order to limit the number of radiomic features that would finally be useful as potential cardiac disease biomarkers, we applied an automated algorithm (see methods) that removed highly correlated features in a stepwise manner. The final set of filtered 84 stable and non-highly correlated radiomic features is presented on a relevant correlation matrix (Fig. 4A, Figure S2).

To explore whether this set of radiomic features could be used to classify cardiac phenotype, we split the cohort into a training (67%) and a validation dataset (33%, Fig. 4B). Then the radiomic features were fed into multinomial logistic regression models which were internally validated by fivefold cross-validation repeated 3 times and the final performance of the model of T1 radiomics to classify cardiac phenotype was explored in the validation dataset. T1 radiomics had a good multinomial logistic c-index of 0.753 for disease classification; a model using only native T1 as a predictor had a poor performance (c-index 0.528). A random forest algorithm was applied to assess the importance of radiomic features and choose the minimum number of features needed to maximize model’s accuracy. A set of 6 radiomic features (LLHglcmImc1, LLHglcmImc2, HLHglszmZoneVariance, Median (first order), LHHglszmLAHGE, LLLglcmMCC) maximized model’s accuracy for cardiac disease classification (Fig. 4C; Table 2). A confusion matrix for the predicted vs. observed classes by the radiomics model in the testing dataset is presented in Fig. 4D). The distribution of the 6 radiomic features among the various disease classes in the testing cohort is shown in Fig. 5A. The added diagnostic value of these 6 radiomic features for cardiac disease phenotypes over and above native T1 alone is shown in relevant ROC curves (Fig. 5B).

**Table 2:** Significance of the top radiomic features included in the final model.

Radiomics	Type	Category	Wavelet	Explanation
First order median	Intensity	First order features	–	The median gray level intensity within the ROI
Zone variance	Texture	Gray level size zone matrix	HLH	Measures the variance in zone size volumes for the zones
Informational measure of correlation (IMC) 1	Texture	Gray level co-occurrence matrix	LLH	Complexity of the texture
Informational measure of correlation (IMC) 2	Texture	Gray level co-occurrence matrix	LLH	Complexity of the texture
LAHGLE (low area high gray level emphasis)	Texture	Gray level size zone matrix	LHH	Measures the proportion in the image of the joint distribution of larger size zones with higher gray-level values
Maximal correlation coefficient (MCC)	Texture	Gray level co-occurrence matrix	LLL	Complexity of the texture

## Discussion

In the present study, we show that radiomic phenotyping of native T1 mapping using machine learning can distinguish healthy vs hypertrophic myocardium and can also differentiate LVH etiology, including HCM or cardiac amyloid. We demonstrated that T1 mapping images of human LV myocardium are a rich source of extractable, quantifiable data. Through a rigorous process that involved machine learning for feature selection and training with internal validation and external testing, we identified 850 distinct T1 radiomic features, that when narrowed down to a subset of six features that outperform mean native T1 values in characterization of myocardial hypertrophy.

The findings of this study expand and re-enforce those of previous studies in the field^10,17^. While thorough analysis of CMR allows for definitive diagnosis, using our approach, T1 mapping can be used as an initial readily available, screening tool for unbiased and standardized myocardial disease detection and classification. Indeed, T1 mapping is a newer approach in the CMR field, which allows detection of alteration in the extracellular matrix of myocardium^18,19^. Various acquisition techniques for T1 mapping have been developed, such as the modified Look Locker inversion recovery (MOLLI)^20^ technique or the shortened MOLLI sequence (ShMOLLI)^21^, and they all allow a standardized acquisition of the T1 relaxation times of the heart and the reconstruction of an image with a well-defined and repeatable relationship between voxel signal intensity and the T1 time^18,19^. T1 mapping allows the detection of subclinical myocardial disease before the development of replacement fibrosis and any LGE presence^22^. Also T1 mapping is useful for the detection of myocardial oedema^23,24^ or even iron overload^25^ and sphingolipid storage disease^26^. The degree of diffuse fibrosis detected by T1 mapping provides not only diagnostic information but also prognostic information as in non-ischaemic cardiomyopathy^27^. Despite this useful clinical information derived from T1 mapping significant overlap exists in native T1 values between health and disease^18,19^. Therefore, unless there is a specific clinical context in which the value of T1 mapping is well-established, T1 mapping is not appropriate as a screening tool for detecting subclinical disease in every patient undergoing a CMR scan. However, our approach provides unique evidence that could enrich the clinical information derived from myocardial T1 maps and further establish the value of T1 mapping in the field of CMR.

Recent advances in biomedical technology and computational power have rendered feasible the application of high-throughput screening of human genome, transcriptome, proteome or metabolome and the acquisition of high-dimensional datasets. Along these domains, radiomics is a rapidly evolving field of medical imaging that relies on high-throughput exploitation of the rich data stored in medical images to extract a series of quantifiable radiomic features. In contrast to other omics technologies that are characterised by high measurement cost and limited temporal applicability, radiomics can be easily applied to detect patterns and extract quantifiable features from standard-of-care medical imaging stacks^5,8^. This approach allows the identification of shape, intensity, and texture patterns in the imaging voxels of interest by application of first, second or higher order statistics^8^. To date radiomics have been successfully applied in the field of clinical oncology, whereby radiomic signatures improve the diagnostic accuracy, staging and grading of cancer, response to treatment and prediction of clinical outcomes^7^. Radiomics have been also applied for the first time in the field of cardiovascular medicine to improve discrimination of high-risk coronary plaques^5^. CMR radiomics from T1 maps has been previously employed to discriminate between hypertensive heart disease and HCM^10^ or even between subtypes of sarcomeric HCM^28^. Our study further expands these findings and shows that radiomics analysis of T1 maps, enhances the value of T1 mapping to discriminate health from disease and classify the various LVH phenotypes. Such evidence suggests that radiomics could contribute to the improvement of clinical diagnostics or to assess the individualised disease risk and response to treatment, and be the key to tailored, personalized medicine^8^.

Our study has certain methodological strengths that lie within the stepwise, rigorous approach we followed. Initially, we demonstrated the proof-of-concept that T1 mapping images can be decomposed to several independent components that describe different aspects of cardiac phenotype and are distinctively associated with the patient profile. Then we focused on 850 different calculated radiomic features to generate radiomic signatures of myocardial health and disease. Since previous evidence suggests that not all T1 radiomics are stable and reproducible^9^, we followed a rigorous stability assessment approach and only those highly reproducible and consistent radiomic features in multiple delineation were used in further exploratory analysis. To facilitate feature selection, we also applied an automated algorithm to remove highly correlated features. Then we explored the value of the 84 retained radiomic features to classify the cardiac phenotype and, ultimately, we demonstrated that a radiomic signature provides incremental value for detection of cardiac disease phenotypes, beyond and above native T1 values.

Certain limitations of our study should be acknowledged. Specifically, this was a proof-of-concept study from a single study centre in which we used only basal T1 map slice for texture analysis, and with a relatively small study population sample. We used the whole cohort to remove highly correlated radiomic features, before training and testing our model. Thus, the generalizability of our findings remains to be seen, and therefore true external validation of our observations from third independent groups, in other scanner types/acquisition settings would be welcome. Given the reproducibility issues of radiomics, further research on the field is needed to harmonize the readouts between different scanners/vendors and acquisition sequences.

## Conclusions

In conclusion, we have presented a novel approach for myocardial texture phenotyping using T1 radiomics analysis. We demonstrated and validated by a machine learning approach that radiomic features provide added diagnostic value for distinction between healthy and diseased myocardium, as well as for differentiation between HCM and relevant phenocopies, such as amyloidosis, on top of native T1. The application of radiomics to standard native T1 mapping is a promising approach for the texture characterization of the human myocardium and for further enhancing the diagnostic value of CMR.

## Supplementary Information

Supplementary Information.
