# Supplementary material for: Machine learning of native T1 mapping radiomics for classification of hypertrophic cardiomyopathy phenotypes
Source: Sci Rep. 2021 Dec 8;11:23596. doi: 10.1038/s41598-021-02971-z (PMC8654857; doi:10.1038/s41598-021-02971-z)
Supplement: Supplementary file 1 — Supplementary Information. [file 41598_2021_2971_MOESM1_ESM.pdf]

ONLINE SUPPLEMENT

Machine learning of native T1 mapping radiomics for classification of hypertrophic cardiomyopathy phenotypes

Alexios S. Antonopoulos[1*], Maria Boutsikou[2*], Spyridon Simantiris[1], Andreas Angelopoulos[1], George Lazaros[1], Ioannis Panagiotopoulos[1], Evangelos Oikonomou[1], Mikela Kanoupaki[2], Dimitris Tousoulis[1], Raad H. Mohiaddin[3], Konstantinos Tsioufis[1], Charalambos Vlachopoulos[1]

[1]Unit of Inherited Cardiac Conditions and Sports Cardiology, 1st Department of Cardiology, National and Kapodistrian University of Athens, Greece

[2]CMR Unit, Mediterraneo Hospital, Attiki, Greece,

[3]CMR Unit, Faculty of Medicine, National Heart and Lung Institute, Imperial College London, UK

Running title: T1 radiomics for classification of cardiac phenotypes

Total word count: 5,297, **Figures**: 5, **Tables**: 2

*equally contributed

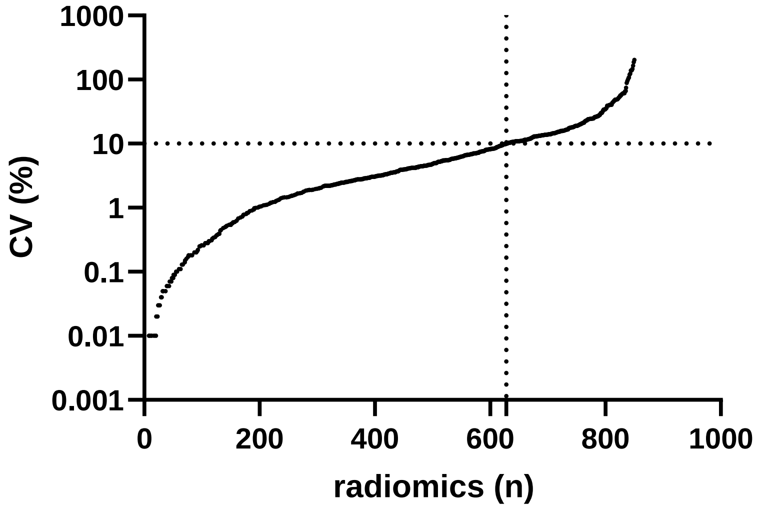

Figure S1. Plot of the number ID of radiomic features vs. coefficient of variation (CV). A total of 628 radiomic features had an inter-observer CV< 10%.

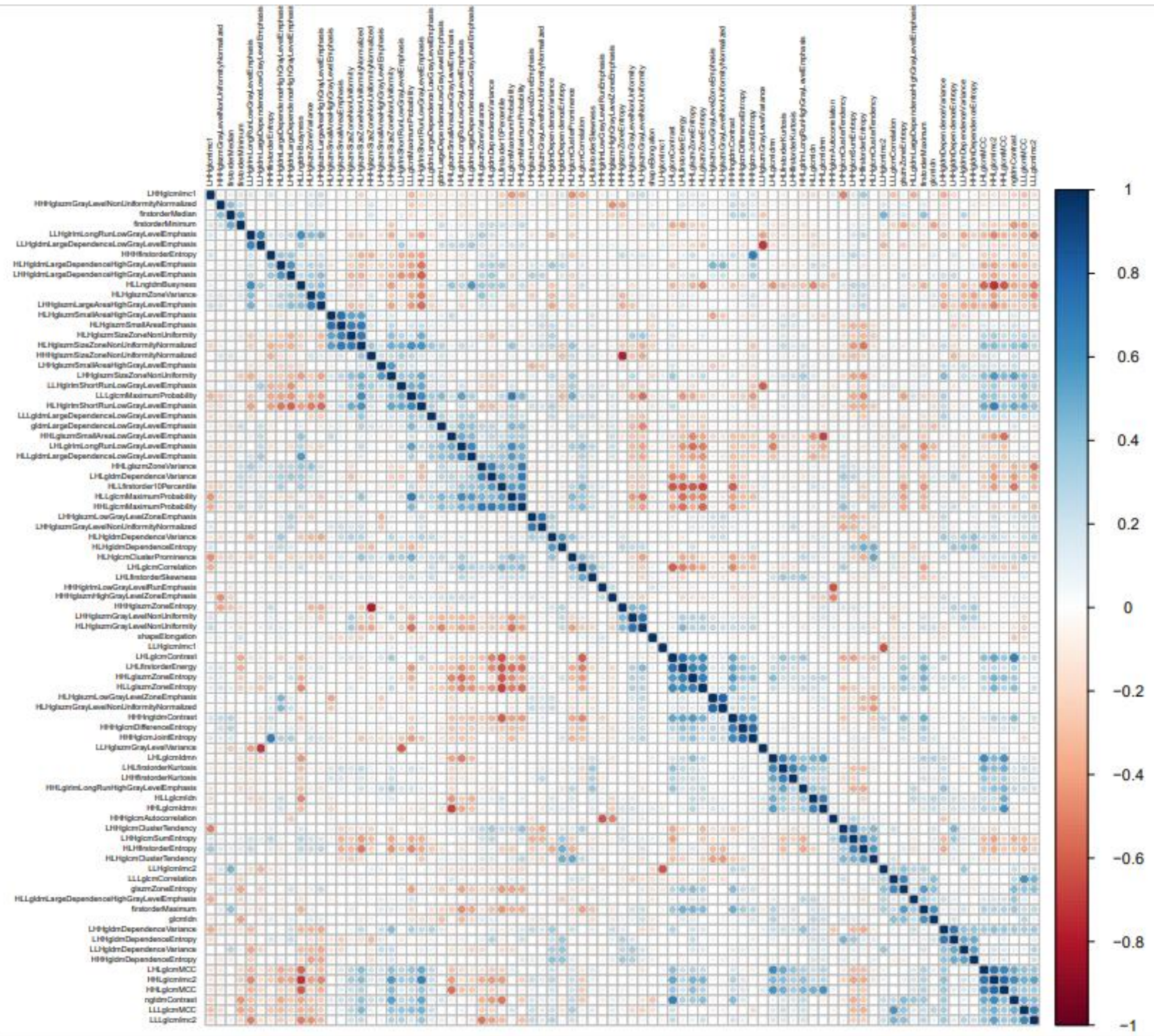

Figure S2. Correlation plot after stepwise exclusion of highly correlated radiomic features (n=84) which were finally entered in the training/validation algorithm. The names of the

List of individual radiomic features and their coefficient of variation.

radiomic features	*CV%*
shapeMaximum3DDiameter	3.03%
shapeMaximum2DDiameterSlice	3.03%
shapeSphericity	1.15%
shapeMinorAxis	0.70%
shapeElongation	1.24%
shapeSurfaceVolumeRatio	2.37%
shapeVolume	3.34%
shapeMajorAxis	1.89%
shapeSurfaceArea	1.13%
shapeMaximum2DDiameterColumn	2.78%
shapeMaximum2DDiameterRow	3.51%
gldmGrayLevelVariance	40.55%
gldmHighGrayLevelEmphasis	13.56%
gldmDependenceEntropy	3.28%
gldmDependenceNonUniformity	3.98%
gldmGrayLevelNonUniformity	13.66%
gldmSmallDependenceEmphasis	0.26%
gldmSmallDependenceHighGrayLevelEmphasis	14.05%
gldmDependenceNonUniformityNormalized	0.68%
gldmLargeDependenceEmphasis	0.89%
gldmLargeDependenceLowGrayLevelEmphasis	1.00%
gldmDependenceVariance	1.22%
gldmLargeDependenceHighGrayLevelEmphasis	14.02%
gldmSmallDependenceLowGrayLevelEmphasis	0.56%
gldmLowGrayLevelEmphasis	0.30%
glcmJointAverage	4.96%
glcmSumAverage	4.96%
glcmJointEntropy	1.14%
glcmClusterShade	108.32%
glcmMaximumProbability	4.41%
glcmIdmn	0.20%
glcmJointEnergy	7.99%
glcmContrast	13.43%
glcmDifferenceEntropy	1.82%
glcmInverseVariance	4.14%
glcmDifferenceVariance	17.65%
glcmIdn	0.75%
glcmIdm	1.70%
glcmCorrelation	6.82%
glcmAutocorrelation	11.80%
glcmSumEntropy	2.86%
glcmMCC	13.69%
glcmSumSquares	29.98%

glcmClusterProminence	106.99%
glcmImc2	1.40%
glcmImc1	-14.50%
glcmDifferenceAverage	4.99%
glcmId	1.44%
glcmClusterTendency	34.01%
firstorderInterquartileRange	13.05%
firstorderSkewness	47.14%
firstorderUniformity	11.05%
firstorderMedian	1.44%
firstorderEnergy	0.18%
firstorderRobustMeanAbsoluteDeviation	14.09%
firstorderMeanAbsoluteDeviation	15.42%
firstorderTotalEnergy	0.18%
firstorderMaximum	8.22%
firstorderRootMeanSquared	1.67%
firstorder90Percentile	2.65%
firstorderMinimum	0.00%
firstorderEntropy	3.65%
firstorderRange	17.90%
firstorderVariance	39.74%
firstorder10Percentile	0.01%
firstorderKurtosis	18.57%
firstorderMean	1.55%
glrlmShortRunLowGrayLevelEmphasis	0.49%
glrlmGrayLevelVariance	40.70%
glrlmLowGrayLevelRunEmphasis	0.44%
glrlmGrayLevelNonUniformityNormalized	10.93%
glrlmRunVariance	4.63%
glrlmGrayLevelNonUniformity	13.59%
glrlmLongRunEmphasis	0.34%
glrlmShortRunHighGrayLevelEmphasis	13.70%
glrlmRunLengthNonUniformity	3.45%
glrlmShortRunEmphasis	0.02%
glrlmLongRunHighGrayLevelEmphasis	13.82%
glrlmRunPercentage	0.07%
glrlmLongRunLowGrayLevelEmphasis	0.30%
glrlmRunEntropy	3.40%
glrlmHighGrayLevelRunEmphasis	13.66%
glrlmRunLengthNonUniformityNormalized	0.04%
glszmGrayLevelVariance	40.70%
glszmZoneVariance	6.72%
glszmGrayLevelNonUniformityNormalized	10.32%
glszmSizeZoneNonUniformityNormalized	0.03%

glszmSizeZoneNonUniformity	3.55%
glszmGrayLevelNonUniformity	13.12%
glszmLargeAreaEmphasis	1.43%
glszmSmallAreaHighGrayLevelEmphasis	14.56%
glszmZonePercentage	0.19%
glszmLargeAreaLowGrayLevelEmphasis	0.30%
glszmLargeAreaHighGrayLevelEmphasis	14.57%
glszmHighGrayLevelZoneEmphasis	14.11%
glszmSmallAreaEmphasis	0.01%
glszmLowGrayLevelZoneEmphasis	0.70%
glszmZoneEntropy	2.92%
glszmSmallAreaLowGrayLevelEmphasis	0.83%
ngtdmCoarseness	10.24%
ngtdmComplexity	48.52%
ngtdmStrength	74.40%
ngtdmContrast	7.55%
ngtdmBusyness	21.26%
HLLgldmGrayLevelVariance	11.81%
HLLgldmHighGrayLevelEmphasis	2.03%
HLLgldmDependenceEntropy	0.16%
HLLgldmDependenceNonUniformity	2.78%
HLLgldmGrayLevelNonUniformity	4.70%
HLLgldmSmallDependenceEmphasis	0.25%
HLLgldmSmallDependenceHighGrayLevelEmphasis	2.03%
HLLgldmDependenceNonUniformityNormalized	0.60%
HLLgldmLargeDependenceEmphasis	2.48%
HLLgldmLargeDependenceLowGrayLevelEmphasis	1.50%
HLLgldmDependenceVariance	7.15%
HLLgldmLargeDependenceHighGrayLevelEmphasis	0.54%
HLLgldmSmallDependenceLowGrayLevelEmphasis	4.88%
HLLgldmLowGrayLevelEmphasis	4.29%
HLLglcmJointAverage	0.99%
HLLglcmSumAverage	0.99%
HLLglcmJointEntropy	0.20%
HLLglcmClusterShade	-56.41%
HLLglcmMaximumProbability	6.64%
HLLglcmIdmn	0.08%
HLLglcmJointEnergy	1.46%
HLLglcmContrast	10.38%
HLLglcmDifferenceEntropy	1.04%
HLLglcmInverseVariance	1.87%
HLLglcmDifferenceVariance	13.85%
HLLglcmIdn	0.28%
HLLglcmIdm	1.91%

HLLglcmCorrelation	10.83%
HLLglcmAutocorrelation	2.36%
HLLglcmSumEntropy	1.23%
HLLglcmMCC	10.99%
HLLglcmSumSquares	15.96%
HLLglcmClusterProminence	65.85%
HLLglcmImc2	0.77%
HLLglcmImc1	-5.51%
HLLglcmDifferenceAverage	3.70%
HLLglcmId	1.51%
HLLglcmClusterTendency	19.01%
HLLfirstorderInterquartileRange	2.94%
HLLfirstorderSkewness	-30.28%
HLLfirstorderUniformity	1.45%
HLLfirstorderMedian	39.29%
HLLfirstorderEnergy	8.25%
HLLfirstorderRobustMeanAbsoluteDeviation	3.17%
HLLfirstorderMeanAbsoluteDeviation	3.98%
HLLfirstorderTotalEnergy	8.25%
HLLfirstorderMaximum	15.71%
HLLfirstorderRootMeanSquared	5.87%
HLLfirstorder90Percentile	2.78%
HLLfirstorderMinimum	0.00%
HLLfirstorderEntropy	0.52%
HLLfirstorderRange	7.53%
HLLfirstorderVariance	11.72%
HLLfirstorder10Percentile	-1.41%
HLLfirstorderKurtosis	12.27%
HLLfirstorderMean	43.32%
HLLglrlmShortRunLowGrayLevelEmphasis	4.37%
HLLglrlmGrayLevelVariance	11.74%
HLLglrlmLowGrayLevelRunEmphasis	4.28%
HLLglrlmGrayLevelNonUniformityNormalized	1.15%
HLLglrlmRunVariance	2.70%
HLLglrlmGrayLevelNonUniformity	4.30%
HLLglrlmLongRunEmphasis	0.34%
HLLglrlmShortRunHighGrayLevelEmphasis	2.10%
HLLglrlmRunLengthNonUniformity	2.95%
HLLglrlmShortRunEmphasis	0.10%
HLLglrlmLongRunHighGrayLevelEmphasis	1.69%
HLLglrlmRunPercentage	0.13%
HLLglrlmLongRunLowGrayLevelEmphasis	3.91%
HLLglrlmRunEntropy	0.26%
HLLglrlmHighGrayLevelRunEmphasis	2.01%

HLLglrlmRunLengthNonUniformityNormalized	0.28%
HLLglszmGrayLevelVariance	11.92%
HLLglszmZoneVariance	0.09%
HLLglszmGrayLevelNonUniformityNormalized	0.50%
HLLglszmSizeZoneNonUniformityNormalized	0.18%
HLLglszmSizeZoneNonUniformity	3.06%
HLLglszmGrayLevelNonUniformity	3.70%
HLLglszmLargeAreaEmphasis	0.20%
HLLglszmSmallAreaHighGrayLevelEmphasis	1.79%
HLLglszmZonePercentage	0.13%
HLLglszmLargeAreaLowGrayLevelEmphasis	3.45%
HLLglszmLargeAreaHighGrayLevelEmphasis	1.76%
HLLglszmHighGrayLevelZoneEmphasis	1.90%
HLLglszmSmallAreaEmphasis	0.08%
HLLglszmLowGrayLevelZoneEmphasis	4.54%
HLLglszmZoneEntropy	0.20%
HLLglszmSmallAreaLowGrayLevelEmphasis	4.83%
HLLngtdmCoarseness	1.80%
HLLngtdmComplexity	20.18%
HLLngtdmStrength	22.36%
HLLngtdmContrast	5.22%
HLLngtdmBusyness	5.51%
LHLgldmGrayLevelVariance	5.49%
LHLgldmHighGrayLevelEmphasis	24.15%
LHLgldmDependenceEntropy	0.07%
LHLgldmDependenceNonUniformity	0.17%
LHLgldmGrayLevelNonUniformity	5.95%
LHLgldmSmallDependenceEmphasis	2.21%
LHLgldmSmallDependenceHighGrayLevelEmphasis	27.93%
LHLgldmDependenceNonUniformityNormalized	3.77%
LHLgldmLargeDependenceEmphasis	2.64%
LHLgldmLargeDependenceLowGrayLevelEmphasis	13.21%
LHLgldmDependenceVariance	0.35%
LHLgldmLargeDependenceHighGrayLevelEmphasis	18.87%
LHLgldmSmallDependenceLowGrayLevelEmphasis	7.58%
LHLgldmLowGrayLevelEmphasis	9.23%
LHLglcmJointAverage	11.58%
LHLglcmSumAverage	11.58%
LHLglcmJointEntropy	0.28%
LHLglcmClusterShade	-16.24%
LHLglcmMaximumProbability	2.54%
LHLglcmIdmn	0.27%
LHLglcmJointEnergy	2.62%
LHLglcmContrast	8.52%

LHLglcmDifferenceEntropy	0.73%
LHLglcmInverseVariance	4.65%
LHLglcmDifferenceVariance	12.28%
LHLglcmIdn	0.62%
LHLglcmIdm	4.17%
LHLglcmCorrelation	2.19%
LHLglcmAutocorrelation	25.40%
LHLglcmSumEntropy	0.71%
LHLglcmMCC	8.38%
LHLglcmSumSquares	7.38%
LHLglcmClusterProminence	17.24%
LHLglcmImc2	1.10%
LHLglcmImc1	-6.44%
LHLglcmDifferenceAverage	3.05%
LHLglcmId	2.30%
LHLglcmClusterTendency	6.75%
LHLfirstorderInterquartileRange	1.58%
LHLfirstorderSkewness	-2.31%
LHLfirstorderUniformity	2.79%
LHLfirstorderMedian	31.93%
LHLfirstorderEnergy	2.47%
LHLfirstorderRobustMeanAbsoluteDeviation	1.94%
LHLfirstorderMeanAbsoluteDeviation	2.25%
LHLfirstorderTotalEnergy	2.47%
LHLfirstorderMaximum	7.04%
LHLfirstorderRootMeanSquared	3.02%
LHLfirstorder90Percentile	6.70%
LHLfirstorderMinimum	-12.92%
LHLfirstorderEntropy	0.79%
LHLfirstorderRange	10.32%
LHLfirstorderVariance	5.82%
LHLfirstorder10Percentile	-0.50%
LHLfirstorderKurtosis	6.92%
LHLfirstorderMean	45.19%
LHLglrlmShortRunLowGrayLevelEmphasis	9.01%
LHLglrlmGrayLevelVariance	5.64%
LHLglrlmLowGrayLevelRunEmphasis	9.20%
LHLglrlmGrayLevelNonUniformityNormalized	3.01%
LHLglrlmRunVariance	3.53%
LHLglrlmGrayLevelNonUniformity	5.93%
LHLglrlmLongRunEmphasis	0.61%
LHLglrlmShortRunHighGrayLevelEmphasis	24.66%
LHLglrlmRunLengthNonUniformity	2.54%
LHLglrlmShortRunEmphasis	0.22%

LHLglrlmLongRunHighGrayLevelEmphasis	23.04%
LHLglrlmRunPercentage	0.26%
LHLglrlmLongRunLowGrayLevelEmphasis	9.88%
LHLglrlmRunEntropy	0.50%
LHLglrlmHighGrayLevelRunEmphasis	24.25%
LHLglrlmRunLengthNonUniformityNormalized	0.59%
LHLglszmGrayLevelVariance	5.99%
LHLglszmZoneVariance	2.55%
LHLglszmGrayLevelNonUniformityNormalized	3.54%
LHLglszmSizeZoneNonUniformityNormalized	3.32%
LHLglszmSizeZoneNonUniformity	1.05%
LHLglszmGrayLevelNonUniformity	5.52%
LHLglszmLargeAreaEmphasis	2.51%
LHLglszmSmallAreaHighGrayLevelEmphasis	26.83%
LHLglszmZonePercentage	1.30%
LHLglszmLargeAreaLowGrayLevelEmphasis	11.89%
LHLglszmLargeAreaHighGrayLevelEmphasis	19.77%
LHLglszmHighGrayLevelZoneEmphasis	24.50%
LHLglszmSmallAreaEmphasis	1.40%
LHLglszmLowGrayLevelZoneEmphasis	9.23%
LHLglszmZoneEntropy	0.22%
LHLglszmSmallAreaLowGrayLevelEmphasis	8.35%
LHLngtdmCoarseness	4.58%
LHLngtdmComplexity	12.72%
LHLngtdmStrength	15.69%
LHLngtdmContrast	1.10%
LHLngtdmBusyness	14.77%
LHHgldmGrayLevelVariance	0.18%
LHHgldmHighGrayLevelEmphasis	0.90%
LHHgldmDependenceEntropy	0.45%
LHHgldmDependenceNonUniformity	4.31%
LHHgldmGrayLevelNonUniformity	3.18%
LHHgldmSmallDependenceEmphasis	7.22%
LHHgldmSmallDependenceHighGrayLevelEmphasis	0.38%
LHHgldmDependenceNonUniformityNormalized	1.04%
LHHgldmLargeDependenceEmphasis	0.26%
LHHgldmLargeDependenceLowGrayLevelEmphasis	2.78%
LHHgldmDependenceVariance	2.76%
LHHgldmLargeDependenceHighGrayLevelEmphasis	2.20%
LHHgldmSmallDependenceLowGrayLevelEmphasis	16.41%
LHHgldmLowGrayLevelEmphasis	0.94%
LHHglcmJointAverage	0.79%
LHHglcmSumAverage	0.79%
LHHglcmJointEntropy	0.34%

LHHglcmClusterShade	-<br>164.85%
LHHglcmMaximumProbability	4.57%
LHHglcmIdmn	0.09%
LHHglcmJointEnergy	0.87%
LHHglcmContrast	1.00%
LHHglcmDifferenceEntropy	0.45%
LHHglcmInverseVariance	1.00%
LHHglcmDifferenceVariance	0.59%
LHHglcmIdn	0.16%
LHHglcmIdm	0.25%
LHHglcmCorrelation	3.56%
LHHglcmAutocorrelation	1.62%
LHHglcmSumEntropy	0.16%
LHHglcmMCC	3.56%
LHHglcmSumSquares	0.18%
LHHglcmClusterProminence	0.54%
LHHglcmImc2	3.17%
LHHglcmImc1	-9.39%
LHHglcmDifferenceAverage	1.00%
LHHglcmId	0.25%
LHHglcmClusterTendency	0.38%
LHHfirstorderInterquartileRange	4.69%
LHHfirstorderSkewness	-50.60%
LHHfirstorderUniformity	0.18%
LHHfirstorderMedian	32.89%
LHHfirstorderEnergy	2.77%
LHHfirstorderRobustMeanAbsoluteDeviation	3.14%
LHHfirstorderMeanAbsoluteDeviation	2.81%
LHHfirstorderTotalEnergy	2.77%
LHHfirstorderMaximum	15.45%
LHHfirstorderRootMeanSquared	3.18%
LHHfirstorder90Percentile	5.45%
LHHfirstorderMinimum	0.00%
LHHfirstorderEntropy	0.13%
LHHfirstorderRange	5.82%
LHHfirstorderVariance	5.93%
LHHfirstorder10Percentile	-1.03%
LHHfirstorderKurtosis	3.06%
LHHfirstorderMean	94.70%
LHHglrlmShortRunLowGrayLevelEmphasis	2.15%
LHHglrlmGrayLevelVariance	0.05%
LHHglrlmLowGrayLevelRunEmphasis	0.31%
LHHglrlmGrayLevelNonUniformityNormalized	0.05%
LHHglrlmRunVariance	2.19%

LHHglrlmGrayLevelNonUniformity	3.13%
LHHglrlmLongRunEmphasis	0.35%
LHHglrlmShortRunHighGrayLevelEmphasis	2.60%
LHHglrlmRunLengthNonUniformity	3.12%
LHHglrlmShortRunEmphasis	0.37%
LHHglrlmLongRunHighGrayLevelEmphasis	1.42%
LHHglrlmRunPercentage	0.28%
LHHglrlmLongRunLowGrayLevelEmphasis	2.20%
LHHglrlmRunEntropy	0.06%
LHHglrlmHighGrayLevelRunEmphasis	0.29%
LHHglrlmRunLengthNonUniformityNormalized	0.18%
LHHglszmGrayLevelVariance	1.98%
LHHglszmZoneVariance	10.88%
LHHglszmGrayLevelNonUniformityNormalized	1.98%
LHHglszmSizeZoneNonUniformityNormalized	0.57%
LHHglszmSizeZoneNonUniformity	2.59%
LHHglszmGrayLevelNonUniformity	5.29%
LHHglszmLargeAreaEmphasis	11.28%
LHHglszmSmallAreaHighGrayLevelEmphasis	4.92%
LHHglszmZonePercentage	7.00%
LHHglszmLargeAreaLowGrayLevelEmphasis	15.73%
LHHglszmLargeAreaHighGrayLevelEmphasis	7.11%
LHHglszmHighGrayLevelZoneEmphasis	6.55%
LHHglszmSmallAreaEmphasis	59.81%
LHHglszmLowGrayLevelZoneEmphasis	6.72%
LHHglszmZoneEntropy	2.37%
LHHglszmSmallAreaLowGrayLevelEmphasis	187.08%
LHHngtdmCoarseness	4.65%
LHHngtdmComplexity	0.98%
LHHngtdmStrength	4.57%
LHHngtdmContrast	1.08%
LHHngtdmBusyness	7.70%
LLHgldmGrayLevelVariance	3.57%
LLHgldmHighGrayLevelEmphasis	0.01%
LLHgldmDependenceEntropy	1.34%
LLHgldmDependenceNonUniformity	6.35%
LLHgldmGrayLevelNonUniformity	3.37%
LLHgldmSmallDependenceEmphasis	4.02%
LLHgldmSmallDependenceHighGrayLevelEmphasis	4.07%
LLHgldmDependenceNonUniformityNormalized	3.22%
LLHgldmLargeDependenceEmphasis	1.69%
LLHgldmLargeDependenceLowGrayLevelEmphasis	1.69%
LLHgldmDependenceVariance	1.10%
LLHgldmLargeDependenceHighGrayLevelEmphasis	1.69%

LLHgldmSmallDependenceLowGrayLevelEmphasis	3.90%
LLHgldmLowGrayLevelEmphasis	0.04%
LLHglcmJointAverage	0.01%
LLHglcmSumAverage	0.01%
LLHglcmJointEntropy	3.02%
LLHglcmClusterShade	-3.98%
LLHglcmMaximumProbability	0.03%
LLHglcmIdmn	0.01%
LLHglcmJointEnergy	0.05%
LLHglcmContrast	4.18%
LLHglcmDifferenceEntropy	2.89%
LLHglcmInverseVariance	4.18%
LLHglcmDifferenceVariance	4.12%
LLHglcmIdn	0.01%
LLHglcmIdm	0.01%
LLHglcmCorrelation	-4.21%
LLHglcmAutocorrelation	0.01%
LLHglcmSumEntropy	2.89%
LLHglcmMCC	4.21%
LLHglcmSumSquares	4.15%
LLHglcmClusterProminence	3.92%
LLHglcmImc2	4.20%
LLHglcmImc1	-5.44%
LLHglcmDifferenceAverage	4.18%
LLHglcmId	0.01%
LLHglcmClusterTendency	4.12%
LLHfirstorderInterquartileRange	3.52%
LLHfirstorderSkewness	120.18%
LLHfirstorderUniformity	0.02%
LLHfirstorderMedian	1.54%
LLHfirstorderEnergy	1.47%
LLHfirstorderRobustMeanAbsoluteDeviation	1.85%
LLHfirstorderMeanAbsoluteDeviation	3.98%
LLHfirstorderTotalEnergy	1.47%
LLHfirstorderMaximum	18.84%
LLHfirstorderRootMeanSquared	2.52%
LLHfirstorder90Percentile	2.04%
LLHfirstorderMinimum	0.00%
LLHfirstorderEntropy	3.03%
LLHfirstorderRange	17.81%
LLHfirstorderVariance	10.82%
LLHfirstorder10Percentile	2.19%
LLHfirstorderKurtosis	12.45%
LLHfirstorderMean	2.11%

LLHglrlmShortRunLowGrayLevelEmphasis	4.08%
LLHglrlmGrayLevelVariance	0.30%
LLHglrlmLowGrayLevelRunEmphasis	0.01%
LLHglrlmGrayLevelNonUniformityNormalized	0.01%
LLHglrlmRunVariance	7.01%
LLHglrlmGrayLevelNonUniformity	0.10%
LLHglrlmLongRunEmphasis	6.25%
LLHglrlmShortRunHighGrayLevelEmphasis	5.62%
LLHglrlmRunLengthNonUniformity	10.89%
LLHglrlmShortRunEmphasis	5.23%
LLHglrlmLongRunHighGrayLevelEmphasis	6.25%
LLHglrlmRunPercentage	3.48%
LLHglrlmLongRunLowGrayLevelEmphasis	6.24%
LLHglrlmRunEntropy	4.44%
LLHglrlmHighGrayLevelRunEmphasis	0.00%
LLHglrlmRunLengthNonUniformityNormalized	10.95%
LLHglszmGrayLevelVariance	4.00%
LLHglszmZoneVariance	19.34%
LLHglszmGrayLevelNonUniformityNormalized	3.20%
LLHglszmSizeZoneNonUniformityNormalized	24.80%
LLHglszmSizeZoneNonUniformity	8.00%
LLHglszmGrayLevelNonUniformity	28.00%
LLHglszmLargeAreaEmphasis	24.37%
LLHglszmSmallAreaHighGrayLevelEmphasis	1.67%
LLHglszmZonePercentage	39.31%
LLHglszmLargeAreaLowGrayLevelEmphasis	24.37%
LLHglszmLargeAreaHighGrayLevelEmphasis	24.37%
LLHglszmHighGrayLevelZoneEmphasis	20.00%
LLHglszmSmallAreaEmphasis	14.58%
LLHglszmLowGrayLevelZoneEmphasis	13.33%
LLHglszmZoneEntropy	54.65%
LLHglszmSmallAreaLowGrayLevelEmphasis	18.65%
LLHngtdmCoarseness	15.09%
LLHngtdmComplexity	8.84%
LLHngtdmStrength	6.82%
LLHngtdmContrast	0.52%
LLHngtdmBusyness	11.57%
HLHgldmGrayLevelVariance	1.07%
HLHgldmHighGrayLevelEmphasis	1.94%
HLHgldmDependenceEntropy	0.09%
HLHgldmDependenceNonUniformity	4.70%
HLHgldmGrayLevelNonUniformity	2.37%
HLHgldmSmallDependenceEmphasis	2.58%
HLHgldmSmallDependenceHighGrayLevelEmphasis	3.71%

HLHgldmDependenceNonUniformityNormalized	1.45%
HLHgldmLargeDependenceEmphasis	3.22%
HLHgldmLargeDependenceLowGrayLevelEmphasis	1.97%
HLHgldmDependenceVariance	6.09%
HLHgldmLargeDependenceHighGrayLevelEmphasis	6.90%
HLHgldmSmallDependenceLowGrayLevelEmphasis	1.53%
HLHgldmLowGrayLevelEmphasis	2.23%
HLHglcmJointAverage	1.32%
HLHglcmSumAverage	1.32%
HLHglcmJointEntropy	1.39%
HLHglcmClusterShade	-34.94%
HLHglcmMaximumProbability	8.04%
HLHglcmIdmn	0.39%
HLHglcmJointEnergy	3.93%
HLHglcmContrast	4.45%
HLHglcmDifferenceEntropy	1.07%
HLHglcmInverseVariance	4.45%
HLHglcmDifferenceVariance	1.47%
HLHglcmIdn	0.69%
HLHglcmIdm	1.12%
HLHglcmCorrelation	13.58%
HLHglcmAutocorrelation	2.83%
HLHglcmSumEntropy	0.59%
HLHglcmMCC	13.58%
HLHglcmSumSquares	1.50%
HLHglcmClusterProminence	2.45%
HLHglcmImc2	13.42%
HLHglcmImc1	-13.86%
HLHglcmDifferenceAverage	4.45%
HLHglcmId	1.12%
HLHglcmClusterTendency	0.53%
HLHfirstorderInterquartileRange	2.25%
HLHfirstorderSkewness	-61.08%
HLHfirstorderUniformity	1.04%
HLHfirstorderMedian	46.97%
HLHfirstorderEnergy	9.86%
HLHfirstorderRobustMeanAbsoluteDeviation	2.89%
HLHfirstorderMeanAbsoluteDeviation	4.02%
HLHfirstorderTotalEnergy	9.86%
HLHfirstorderMaximum	28.67%
HLHfirstorderRootMeanSquared	6.64%
HLHfirstorder90Percentile	4.47%
HLHfirstorderMinimum	-2.53%
HLHfirstorderEntropy	0.78%

HLHfirstorderRange	15.26%
HLHfirstorderVariance	13.68%
HLHfirstorder10Percentile	-0.15%
HLHfirstorderKurtosis	16.19%
HLHfirstorderMean	35.91%
HLHglrlmShortRunLowGrayLevelEmphasis	0.63%
HLHglrlmGrayLevelVariance	0.06%
HLHglrlmLowGrayLevelRunEmphasis	0.48%
HLHglrlmGrayLevelNonUniformityNormalized	0.06%
HLHglrlmRunVariance	15.46%
HLHglrlmGrayLevelNonUniformity	4.80%
HLHglrlmLongRunEmphasis	6.35%
HLHglrlmShortRunHighGrayLevelEmphasis	3.29%
HLHglrlmRunLengthNonUniformity	7.46%
HLHglrlmShortRunEmphasis	1.91%
HLHglrlmLongRunHighGrayLevelEmphasis	11.11%
HLHglrlmRunPercentage	1.62%
HLHglrlmLongRunLowGrayLevelEmphasis	0.10%
HLHglrlmRunEntropy	0.51%
HLHglrlmHighGrayLevelRunEmphasis	0.47%
HLHglrlmRunLengthNonUniformityNormalized	2.48%
HLHglszmGrayLevelVariance	2.68%
HLHglszmZoneVariance	8.41%
HLHglszmGrayLevelNonUniformityNormalized	2.42%
HLHglszmSizeZoneNonUniformityNormalized	2.81%
HLHglszmSizeZoneNonUniformity	7.63%
HLHglszmGrayLevelNonUniformity	2.93%
HLHglszmLargeAreaEmphasis	6.08%
HLHglszmSmallAreaHighGrayLevelEmphasis	6.86%
HLHglszmZonePercentage	1.88%
HLHglszmLargeAreaLowGrayLevelEmphasis	7.61%
HLHglszmLargeAreaHighGrayLevelEmphasis	5.53%
HLHglszmHighGrayLevelZoneEmphasis	3.25%
HLHglszmSmallAreaEmphasis	4.48%
HLHglszmLowGrayLevelZoneEmphasis	2.48%
HLHglszmZoneEntropy	1.88%
HLHglszmSmallAreaLowGrayLevelEmphasis	2.61%
HLHngtdmCoarseness	8.80%
HLHngtdmComplexity	4.44%
HLHngtdmStrength	8.56%
HLHngtdmContrast	5.23%
HLHngtdmBusyness	13.13%
HHHgldmGrayLevelVariance	0.05%
HHHgldmHighGrayLevelEmphasis	0.54%

HHHgldmDependenceEntropy	0.84%
HHHgldmDependenceNonUniformity	5.21%
HHHgldmGrayLevelNonUniformity	3.39%
HHHgldmSmallDependenceEmphasis	9.49%
HHHgldmSmallDependenceHighGrayLevelEmphasis	10.92%
HHHgldmDependenceNonUniformityNormalized	2.00%
HHHgldmLargeDependenceEmphasis	2.46%
HHHgldmLargeDependenceLowGrayLevelEmphasis	2.68%
HHHgldmDependenceVariance	4.21%
HHHgldmLargeDependenceHighGrayLevelEmphasis	2.23%
HHHgldmSmallDependenceLowGrayLevelEmphasis	8.08%
HHHgldmLowGrayLevelEmphasis	0.52%
HHHglcmJointAverage	0.11%
HHHglcmSumAverage	0.11%
HHHglcmJointEntropy	0.10%
HHHglcmClusterShade	18.23%
HHHglcmMaximumProbability	1.45%
HHHglcmIdmn	0.11%
HHHglcmJointEnergy	0.27%
HHHglcmContrast	1.20%
HHHglcmDifferenceEntropy	0.19%
HHHglcmInverseVariance	1.20%
HHHglcmDifferenceVariance	0.26%
HHHglcmIdn	0.20%
HHHglcmIdm	0.33%
HHHglcmCorrelation	8.13%
HHHglcmAutocorrelation	0.10%
HHHglcmSumEntropy	0.21%
HHHglcmMCC	8.13%
HHHglcmSumSquares	0.01%
HHHglcmClusterProminence	0.92%
HHHglcmImc2	8.07%
HHHglcmImc1	-13.00%
HHHglcmDifferenceAverage	1.20%
HHHglcmId	0.33%
HHHglcmClusterTendency	0.91%
HHHfirstorderInterquartileRange	5.45%
HHHfirstorderSkewness	-123.94%
HHHfirstorderUniformity	0.05%
HHHfirstorderMedian	-22.64%
HHHfirstorderEnergy	15.97%
HHHfirstorderRobustMeanAbsoluteDeviation	4.38%
HHHfirstorderMeanAbsoluteDeviation	6.21%
HHHfirstorderTotalEnergy	15.97%

HHHfirstorderMaximum	88.78%
HHHfirstorderRootMeanSquared	9.45%
HHHfirstorder90Percentile	5.69%
HHHfirstorderMinimum	-9.69%
HHHfirstorderEntropy	0.03%
HHHfirstorderRange	23.13%
HHHfirstorderVariance	20.91%
HHHfirstorder10Percentile	-7.00%
HHHfirstorderKurtosis	40.63%
HHHfirstorderMean	-34.25%
HHHglrlmShortRunLowGrayLevelEmphasis	0.07%
HHHglrlmGrayLevelVariance	0.05%
HHHglrlmLowGrayLevelRunEmphasis	0.66%
HHHglrlmGrayLevelNonUniformityNormalized	0.05%
HHHglrlmRunVariance	5.97%
HHHglrlmGrayLevelNonUniformity	1.96%
HHHglrlmLongRunEmphasis	3.73%
HHHglrlmShortRunHighGrayLevelEmphasis	2.31%
HHHglrlmRunLengthNonUniformity	1.96%
HHHglrlmShortRunEmphasis	1.09%
HHHglrlmLongRunHighGrayLevelEmphasis	2.73%
HHHglrlmRunPercentage	1.53%
HHHglrlmLongRunLowGrayLevelEmphasis	4.68%
HHHglrlmRunEntropy	0.38%
HHHglrlmHighGrayLevelRunEmphasis	0.69%
HHHglrlmRunLengthNonUniformityNormalized	0.02%
HHHglszmGrayLevelVariance	0.11%
HHHglszmZoneVariance	21.27%
HHHglszmGrayLevelNonUniformityNormalized	0.11%
HHHglszmSizeZoneNonUniformityNormalized	7.17%
HHHglszmSizeZoneNonUniformity	19.53%
HHHglszmGrayLevelNonUniformity	10.68%
HHHglszmLargeAreaEmphasis	20.92%
HHHglszmSmallAreaHighGrayLevelEmphasis	52.29%
HHHglszmZonePercentage	15.17%
HHHglszmLargeAreaLowGrayLevelEmphasis	19.15%
HHHglszmLargeAreaHighGrayLevelEmphasis	22.73%
HHHglszmHighGrayLevelZoneEmphasis	0.41%
HHHglszmSmallAreaEmphasis	56.26%
HHHglszmLowGrayLevelZoneEmphasis	0.45%
HHHglszmZoneEntropy	1.21%
HHHglszmSmallAreaLowGrayLevelEmphasis	61.50%
HHHngtdmCoarseness	1.91%
HHHngtdmComplexity	1.61%

HHHngtdmStrength	1.90%
HHHngtdmContrast	1.67%
HHHngtdmBusyness	4.54%
HHLgldmGrayLevelVariance	26.07%
HHLgldmHighGrayLevelEmphasis	8.47%
HHLgldmDependenceEntropy	0.93%
HHLgldmDependenceNonUniformity	2.40%
HHLgldmGrayLevelNonUniformity	7.01%
HHLgldmSmallDependenceEmphasis	0.54%
HHLgldmSmallDependenceHighGrayLevelEmphasis	7.48%
HHLgldmDependenceNonUniformityNormalized	1.01%
HHLgldmLargeDependenceEmphasis	1.54%
HHLgldmLargeDependenceLowGrayLevelEmphasis	11.02%
HHLgldmDependenceVariance	2.42%
HHLgldmLargeDependenceHighGrayLevelEmphasis	10.55%
HHLgldmSmallDependenceLowGrayLevelEmphasis	10.86%
HHLgldmLowGrayLevelEmphasis	11.19%
HHLglcmJointAverage	5.34%
HHLglcmSumAverage	5.34%
HHLglcmJointEntropy	1.29%
HHLglcmClusterShade	-146.91%
HHLglcmMaximumProbability	2.78%
HHLglcmIdmn	0.18%
HHLglcmJointEnergy	4.17%
HHLglcmContrast	30.03%
HHLglcmDifferenceEntropy	2.68%
HHLglcmInverseVariance	1.74%
HHLglcmDifferenceVariance	44.24%
HHLglcmIdn	0.84%
HHLglcmIdm	1.22%
HHLglcmCorrelation	12.89%
HHLglcmAutocorrelation	10.05%
HHLglcmSumEntropy	1.85%
HHLglcmMCC	5.48%
HHLglcmSumSquares	26.68%
HHLglcmClusterProminence	140.73%
HHLglcmImc2	5.61%
HHLglcmImc1	-17.90%
HHLglcmDifferenceAverage	7.22%
HHLglcmId	1.11%
HHLglcmClusterTendency	24.15%
HHLfirstorderInterquartileRange	5.51%
HHLfirstorderSkewness	-203.97%

HHLfirstorderUniformity	3.93%
HHLfirstorderMedian	-6.10%
HHLfirstorderEnergy	20.51%
HHLfirstorderRobustMeanAbsoluteDeviation	3.88%
HHLfirstorderMeanAbsoluteDeviation	6.63%
HHLfirstorderTotalEnergy	20.51%
HHLfirstorderMaximum	58.53%
HHLfirstorderRootMeanSquared	11.47%
HHLfirstorder90Percentile	5.01%
HHLfirstorderMinimum	-5.16%
HHLfirstorderEntropy	2.06%
HHLfirstorderRange	24.58%
HHLfirstorderVariance	25.79%
HHLfirstorder10Percentile	-14.85%
HHLfirstorderKurtosis	66.18%
HHLfirstorderMean	-10.55%
HHLglrlmShortRunLowGrayLevelEmphasis	10.91%
HHLglrlmGrayLevelVariance	26.11%
HHLglrlmLowGrayLevelRunEmphasis	10.98%
HHLglrlmGrayLevelNonUniformityNormalized	3.61%
HHLglrlmRunVariance	4.16%
HHLglrlmGrayLevelNonUniformity	6.58%
HHLglrlmLongRunEmphasis	0.64%
HHLglrlmShortRunHighGrayLevelEmphasis	8.26%
HHLglrlmRunLengthNonUniformity	3.14%
HHLglrlmShortRunEmphasis	0.04%
HHLglrlmLongRunHighGrayLevelEmphasis	9.18%
HHLglrlmRunPercentage	0.15%
HHLglrlmLongRunLowGrayLevelEmphasis	10.87%
HHLglrlmRunEntropy	1.64%
HHLglrlmHighGrayLevelRunEmphasis	8.37%
HHLglrlmRunLengthNonUniformityNormalized	0.08%
HHLglszmGrayLevelVariance	26.28%
HHLglszmZoneVariance	4.98%
HHLglszmGrayLevelNonUniformityNormalized	2.87%
HHLglszmSizeZoneNonUniformityNormalized	0.03%
HHLglszmSizeZoneNonUniformity	2.63%
HHLglszmGrayLevelNonUniformity	5.32%
HHLglszmLargeAreaEmphasis	2.87%
HHLglszmSmallAreaHighGrayLevelEmphasis	7.81%
HHLglszmZonePercentage	0.80%
HHLglszmLargeAreaLowGrayLevelEmphasis	9.07%
HHLglszmLargeAreaHighGrayLevelEmphasis	11.42%
HHLglszmHighGrayLevelZoneEmphasis	8.09%

HHLglszmSmallAreaEmphasis	0.00%
HHLglszmLowGrayLevelZoneEmphasis	10.24%
HHLglszmZoneEntropy	0.89%
HHLglszmSmallAreaLowGrayLevelEmphasis	9.89%
HHLngtdmCoarseness	3.34%
HHLngtdmComplexity	39.70%
HHLngtdmStrength	53.27%
HHLngtdmContrast	11.50%
HHLngtdmBusyness	7.61%
LLLgldmGrayLevelVariance	49.15%
LLLgldmHighGrayLevelEmphasis	16.39%
LLLgldmDependenceEntropy	1.93%
LLLgldmDependenceNonUniformity	2.16%
LLLgldmGrayLevelNonUniformity	13.33%
LLLgldmSmallDependenceEmphasis	0.56%
LLLgldmSmallDependenceHighGrayLevelEmphasis	17.77%
LLLgldmDependenceNonUniformityNormalized	1.27%
LLLgldmLargeDependenceEmphasis	1.56%
LLLgldmLargeDependenceLowGrayLevelEmphasis	2.26%
LLLgldmDependenceVariance	4.55%
LLLgldmLargeDependenceHighGrayLevelEmphasis	12.73%
LLLgldmSmallDependenceLowGrayLevelEmphasis	2.29%
LLLgldmLowGrayLevelEmphasis	2.30%
LLLglcmJointAverage	5.83%
LLLglcmSumAverage	5.83%
LLLglcmJointEntropy	0.06%
LLLglcmClusterShade	139.53%
LLLglcmMaximumProbability	5.10%
LLLglcmIdmn	0.22%
LLLglcmJointEnergy	0.91%
LLLglcmContrast	12.89%
LLLglcmDifferenceEntropy	1.24%
LLLglcmInverseVariance	1.45%
LLLglcmDifferenceVariance	14.40%
LLLglcmIdn	0.87%
LLLglcmIdm	2.99%
LLLglcmCorrelation	6.88%
LLLglcmAutocorrelation	14.19%
LLLglcmSumEntropy	2.17%
LLLglcmMCC	6.11%
LLLglcmSumSquares	35.41%
LLLglcmClusterProminence	139.13%
LLLglcmImc2	0.07%
LLLglcmImc1	-8.84%

LLLglcmDifferenceAverage	5.50%
LLLglcmId	2.89%
LLLglcmClusterTendency	39.97%
LLLfirstorderInterquartileRange	18.07%
LLLfirstorderSkewness	61.39%
LLLfirstorderUniformity	10.70%
LLLfirstorderMedian	1.26%
LLLfirstorderEnergy	0.13%
LLLfirstorderRobustMeanAbsoluteDeviation	18.11%
LLLfirstorderMeanAbsoluteDeviation	18.93%
LLLfirstorderTotalEnergy	0.13%
LLLfirstorderMaximum	10.11%
LLLfirstorderRootMeanSquared	1.70%
LLLfirstorder90Percentile	3.17%
LLLfirstorderMinimum	0.00%
LLLfirstorderEntropy	2.74%
LLLfirstorderRange	23.96%
LLLfirstorderVariance	48.95%
LLLfirstorder10Percentile	0.03%
LLLfirstorderKurtosis	27.19%
LLLfirstorderMean	1.58%
LLLglrlmShortRunLowGrayLevelEmphasis	2.20%
LLLglrlmGrayLevelVariance	49.11%
LLLglrlmLowGrayLevelRunEmphasis	2.20%
LLLglrlmGrayLevelNonUniformityNormalized	10.69%
LLLglrlmRunVariance	6.48%
LLLglrlmGrayLevelNonUniformity	13.25%
LLLglrlmLongRunEmphasis	0.30%
LLLglrlmShortRunHighGrayLevelEmphasis	16.64%
LLLglrlmRunLengthNonUniformity	3.12%
LLLglrlmShortRunEmphasis	0.06%
LLLglrlmLongRunHighGrayLevelEmphasis	15.81%
LLLglrlmRunPercentage	0.09%
LLLglrlmLongRunLowGrayLevelEmphasis	2.20%
LLLglrlmRunEntropy	2.57%
LLLglrlmHighGrayLevelRunEmphasis	16.48%
LLLglrlmRunLengthNonUniformityNormalized	0.14%
LLLglszmGrayLevelVariance	48.95%
LLLglszmZoneVariance	5.76%
LLLglszmGrayLevelNonUniformityNormalized	10.64%
LLLglszmSizeZoneNonUniformityNormalized	0.69%
LLLglszmSizeZoneNonUniformity	2.33%
LLLglszmGrayLevelNonUniformity	12.99%
LLLglszmLargeAreaEmphasis	1.13%

LLLglszmSmallAreaHighGrayLevelEmphasis	17.51%
LLLglszmZonePercentage	0.39%
LLLglszmLargeAreaLowGrayLevelEmphasis	1.86%
LLLglszmLargeAreaHighGrayLevelEmphasis	14.29%
LLLglszmHighGrayLevelZoneEmphasis	16.78%
LLLglszmSmallAreaEmphasis	0.28%
LLLglszmLowGrayLevelZoneEmphasis	1.89%
LLLglszmZoneEntropy	2.19%
LLLglszmSmallAreaLowGrayLevelEmphasis	1.89%
LLLngtdmCoarseness	9.87%
LLLngtdmComplexity	34.20%
LLLngtdmStrength	60.27%
LLLngtdmContrast	26.83%
LLLngtdmBusyness	14.82%
